# Febuxostat, a Xanthine Oxidase Inhibitor, Decreased Macrophage Matrix Metalloproteinase Expression in Hypoxia

**DOI:** 10.3390/biomedicines8110470

**Published:** 2020-11-03

**Authors:** Shuoyu Wei, Takayuki Isagawa, Masamichi Eguchi, Daisuke Sato, Hiroto Tsukano, Keishi Miyata, Yuichi Oike, Norihiko Takeda, Satoshi Ikeda, Hiroaki Kawano, Koji Maemura

**Affiliations:** 1 Department of Cardiovascular Medicine, Nagasaki University Graduate School of Biomedical Sciences, Sakamoto, Nagasaki 852-8501, Japan; bb55316008@ms.nagasaki-u.ac.jp (S.W.); masa5555@nagasaki-u.ac.jp (M.E.); daisukes@nagasaki-u.ac.jp (D.S.); sikeda@nagasaki-u.ac.jp (S.I.); hkawano@nagasaki-u.ac.jp (H.K.); 2 Center for Data Science, Jichi Medical University, Shimotsuke, Tochigi 329-0498, Japan; 3 Department of Molecular Genetics, Graduate School of Medical Sciences, Kumamoto University, Chuo-ku, Kumamoto 860-8556, Japan; chukano@sings.jp (H.T.); hully@gpo.kumamoto-u.ac.jp (K.M.); oike@gpo.kumamoto-u.ac.jp (Y.O.); 4 Division of Cardiology and Metabolism, Center for Molecular Medicine, Jichi Medical University, Shimotsuke, Tochigi 329-0498, Japan; ntakeda-tky@jichi.ac.jp

**Keywords:** hypoxia, macrophage, matrix metalloproteinase, atherosclerotic plaque rupture, oxidative stress, xanthine oxidase

## Abstract

Macrophages in the atheroma region produce matrix metalloproteinases (MMPs) and decrease plaque stability. Tissue oxygen tension decreases in the arterial wall of the atherosclerotic region. Hypoxia inducible factor (HIF)-1α plays a critical role in the transcriptional activation of hypoxia inducible genes. However, the precise roles of HIF-1α independent pathways in hypoxic responses are largely unknown. Xanthine oxidase (XO) is an enzyme that utilizes molecular oxygen and produces reactive oxygen species (ROS). Here, we show that ROS derived from XO increases *MMP*-3, -10, and -13 expression in murine macrophages. We found that the transcript levels of macrophage *MMP*-3, -10, and -13 were increased in hypoxic conditions. Hypoxia induced MMP expression in HIF-1α deficient macrophages. *N*-acetylcysteine (NAC) or febuxostat, an XO inhibitor, suppressed MMP expression in murine macrophages. Febuxostat decreased the incidence of plaque rupture in apolipoprotein-E-deficient mice. Our results indicate that febuxostat stabilized atherosclerotic plaque via suppressing the activities of macrophage MMP-9 and -13. Febuxostat administration is a potential therapeutic option in the management of atherosclerotic patients.

## 1. Introduction

The incidence rate of cardiovascular events, including ischemic heart diseases and stroke, is increasing annually worldwide. Thrombosis caused by atherosclerotic plaque rupture leads to acute myocardial infarction, unstable angina, and sudden death from ischemic heart disease, known as acute coronary syndrome [1,2,3]. Therefore, it is important to understand the mechanisms of atherosclerotic plaque vulnerability and rupture to prevent acute coronary syndrome. The features of plaque vulnerability include a large necrotic core, high infiltration of inflammatory macrophages, and a thin fibrous cap composed of a small number of collagen fibers. Macrophages are immune cells abundant in atherosclerotic lesions, and they play pivotal roles in the initiation and progression of atherosclerosis. Plaque macrophages express matrix metalloproteinases (MMPs) and weaken the fibrous cap of plaque by degrading extracellular matrix molecules [4,5]. Plaques with a weakened fibrous cap may rupture and cause thrombosis.

Hypoxic environments in plaque lesions have been shown to accelerate atherosclerosis progression [6,7]. The arterial wall of an atherosclerotic plaque is thickened as atherosclerosis progresses; therefore, the diffusion efficiency of oxygen is reduced [8]. In parallel, the oxygen consumption within atherosclerotic plaque lesions is increased by the metabolic activation of infiltrated immune cells, leading to hypoxia [9,10]. It has been suggested that hypoxia activates the expression and secretion of MMPs in atherosclerotic lesions and contributes to plaque instability by degrading extracellular matrix proteins [7,11]. Hypoxia has been shown to produce reactive oxygen species (ROS) [12,13,14,15], leading to the development of atherosclerosis, ischemia-reperfusion injury, cardiomyopathy, and heart failure. Hypoxia inducible factor (HIF)-1α plays an integral role during the transcriptional response to hypoxia [16]. We and others have previously shown that HIF signaling significantly modulates macrophage functions, including cytokine production and migratory capacity [17,18,19]. Xanthine oxidase (XO) is an oxygen-consuming enzyme that oxidizes hypoxanthine or xanthine and produces ROS. While local oxygen tension could affect XO activity and ROS production, the roles of XO in macrophage function are not yet fully elucidated.

In this study, we tested the roles of XO in macrophage activation using an XO inhibitor, febuxostat. Febuxostat suppressed hypoxic induction of *MMP*-3, -10, and -13 in murine macrophages. Furthermore, febuxostat reduced the incidence of atherosclerotic plaque rupture in apolipoprotein-E-(apoE) deficient mice. These results demonstrate that febuxostat administration is a potential therapeutic option in the management of atheromatous patients as it suppresses macrophage MMP activities.

## 2. Results

### 2.1. Hypoxic Stimulation-Induced mRNA Expression of MMP-3, -10, and -13 in Thioglycollate-Elicited Peritoneal Macrophages (TEPMs) in a HIF-1α-Independent Manner

**Figure 1:**
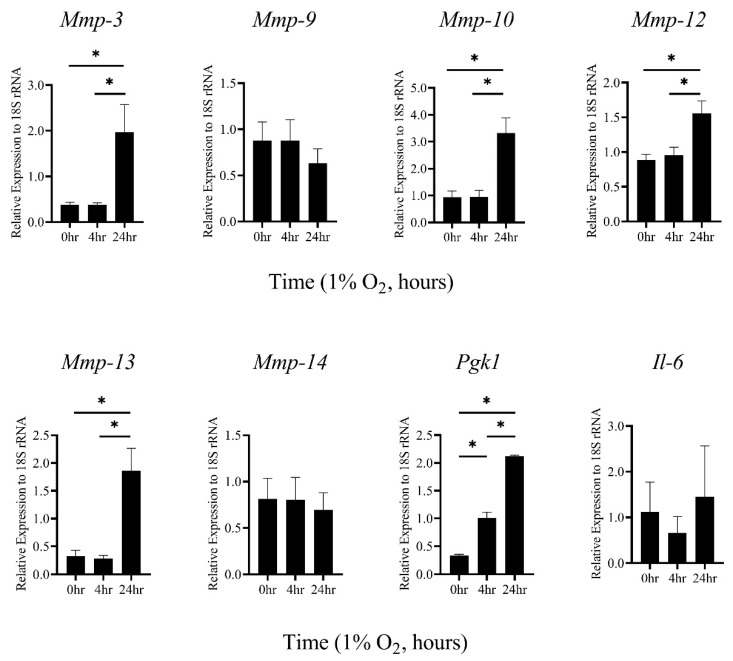
*MMP*-3, -10, and -13 mRNAs in TEPMs are induced by hypoxia. TEPMs were exposed to hypoxia (1% O_2_), and the relative expression level of *MMPs* mRNAs was analyzed. Data are the means and SE of at least three independent experiments. The one-way ANOVA and Tukey’s multiple comparisons test were performed for the statistical analysis. * *p* < 0.05.

**Figure 2:**
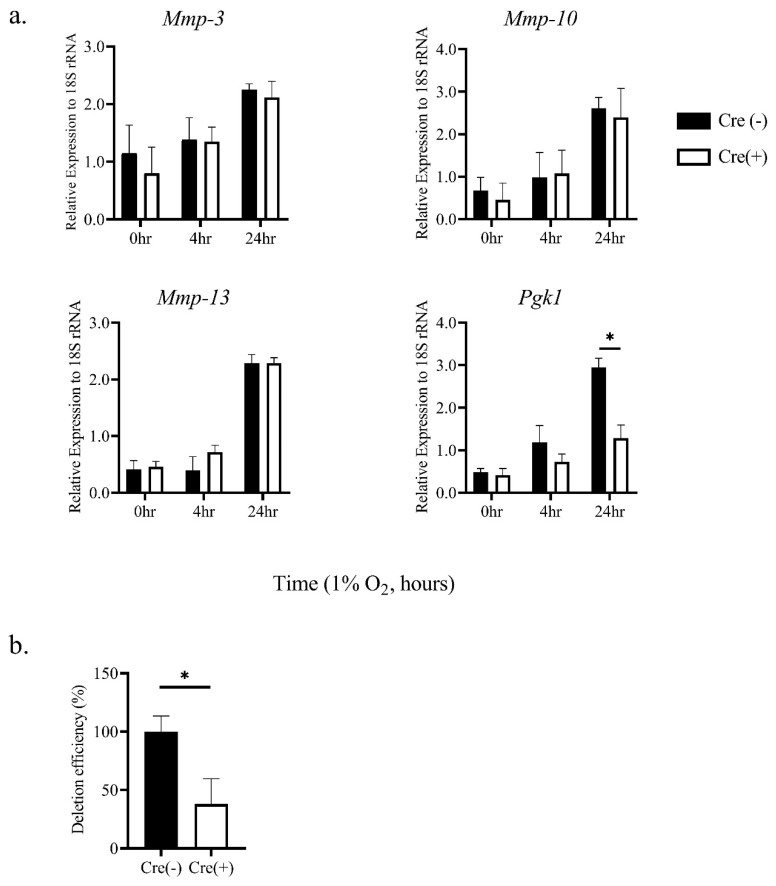
Hypoxic induction of *MMP*-3, -10, and -13 mRNAs is independent of HIF-1α activity. (**a**) TEPMs were isolated from hematopoietic/endothelial-specific *HIF*-*1α* knockout mice (Cre (+)) or cre-negative littermates (Cre (-)) as a control. TEPMs were exposed to hypoxia (1% O_2_), and the relative expression levels of *MMP*-3, -10, and -13 mRNAs were analyzed. Quantitative PCR analysis was repeated in at least three independent experiments. The two-way ANOVA and Tukey’s multiple comparisons test were performed for the statistical analysis. * *p* < 0.05. (**b**) The deletion efficiency of *HIF*-1α mRNA in TEPMs was examined. Data are the means and SE of at least three independent experiments. The unpaired *t*-test was performed for the statistical analysis. * *p* < 0.05.

Macrophages in atherosclerotic plaque are exposed to hypoxic conditions [20]. Therefore, to examine the expression level of various *MMP* mRNAs under hypoxic conditions (1% oxygen concentration), primary TEPMs were isolated from mice. It was found that mRNAs of *MMP*-3, -10, and -13 were significantly increased under hypoxia (Figure 1), whereas the expression of other *MMPs* was unaffected (Figure 1 and data not shown). HIF-1α is the central regulator of the cellular response to hypoxia. Thus, to examine whether HIF-1α was involved in hypoxia-induced elevation of these *MMP* gene expressions, primary TEPMs were isolated from hematopoietic/endothelial-specific *HIF*-1*α* knockout mice (*HIF*-1*α^flox^*^/*flox*^; *Tie*2-*cre*^+/−^ mice; *HIF*-1*α* KO) or cre-negative littermate controls. *Pgk*1 mRNA, well-known as a HIF-1α target gene, was significantly reduced in *HIF*-1*α* KO TEPMs (Figure 2a). However, *MMP*-3, -10, and -13 mRNAs were induced under hypoxic conditions in *HIF*-1*α* KO TEPMs, as in wild-type TEPMs (Figure 2a,b). These results showed that hypoxia increased *MMP*-3, -10, and -13 mRNAs in a HIF-1α-independent manner.

### 2.2. The Induction of MMP-3 and -10 mRNAs by Hypoxia Was Dependent on ROS Generated through XO Activity

**Figure 3:**
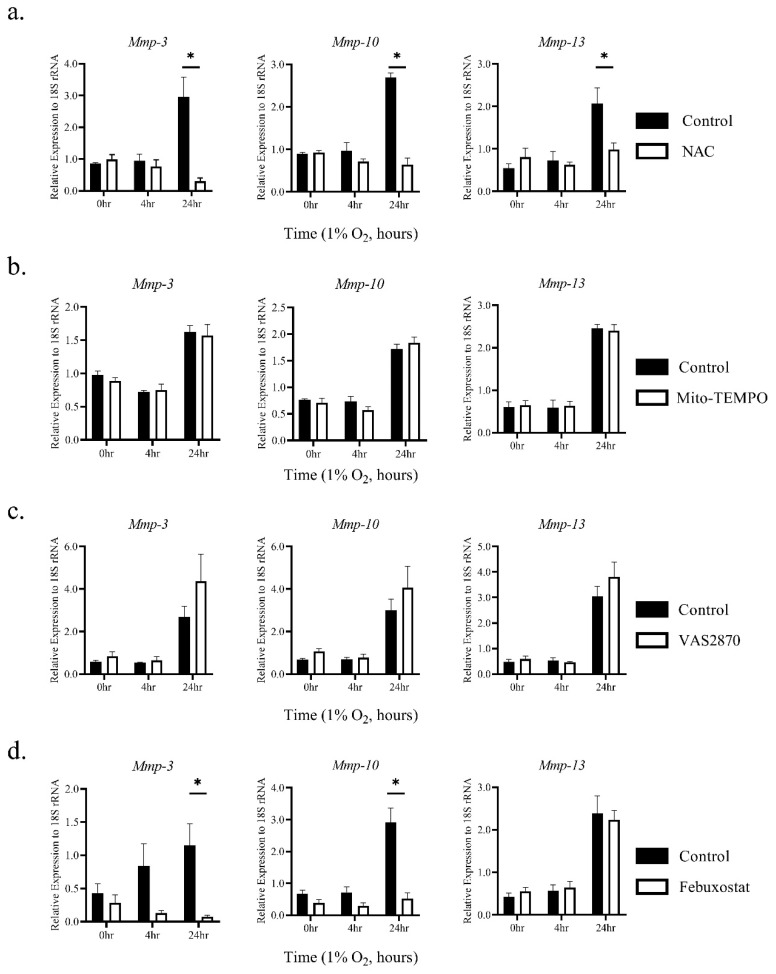
Hypoxia induces *MMP*-3 and *MMP*-10 mRNAs in TEPMs through reactive oxygen species (ROS) derived from xanthine oxidase (XO). TEPMs were exposed to hypoxia (1% O_2_) after pretreatment with dimethyl sulfoxide (DMSO) (control), (**a**) 10 mM *N*-Acetyl-l-cysteine (NAC) (anti-oxidant), (**b**) 50 μM Mito-TEMPO, (**c**) 2 μM VAS2870, or (**d**) 50 μM febuxostat for 1 h. Data are the means and SE of at least three independent experiments. The two-way ANOVA and Tukey’s multiple comparisons test were performed for the statistical analysis. * *p* < 0.05.

Cells cultured under hypoxic conditions have elevated intracellular ROS [12,21]. We hypothesized that such elevated ROS in TEPMs might be responsible for the increased expression of *MMP*-3, -10, and -13 mRNAs. To test this, we asked whether the ROS scavenger *N*-acetyl-l-cysteine (NAC) could attenuate the expression of these MMPs under hypoxic conditions. The treatment with NAC significantly suppressed hypoxic elevation of *MMP*-3, -10, and -13 mRNAs (Figure 3a). It is well-known that intracellular ROS is mainly derived from mitochondria, NADPH oxidase, and xanthine oxidase (XO) [22,23]. To identify which ROS sources were involved in the up-regulation of *MMP*-3, -10, and -13 mRNAs under hypoxic conditions, selective inhibitions of mitochondrial ROS with Mito-TEMPO, NADPH oxidase with VAS2870, and XO with febuxostat were performed. While *MMP*-13 was not suppressed by any inhibitors, hypoxic induction of *MMP*-3 and -10 mRNAs was significantly suppressed by febuxostat, but not Mito-TEMPO and VAS2870 (Figure 3b–d). Moreover, ROS signaling by XO under hypoxia did not affect the accumulation and transcriptional activity of HIF-1α protein (Supplementary Figure S1a–c). These findings suggest that ROS derived from XO contributes to the hypoxia-induced elevation of *MMP*-3 and -10 mRNAs in TEPMs independent of HIF-1α signaling.

### 2.3. Hypoxia Activates MMPs via ROS Derived from XO

**Figure 4:**
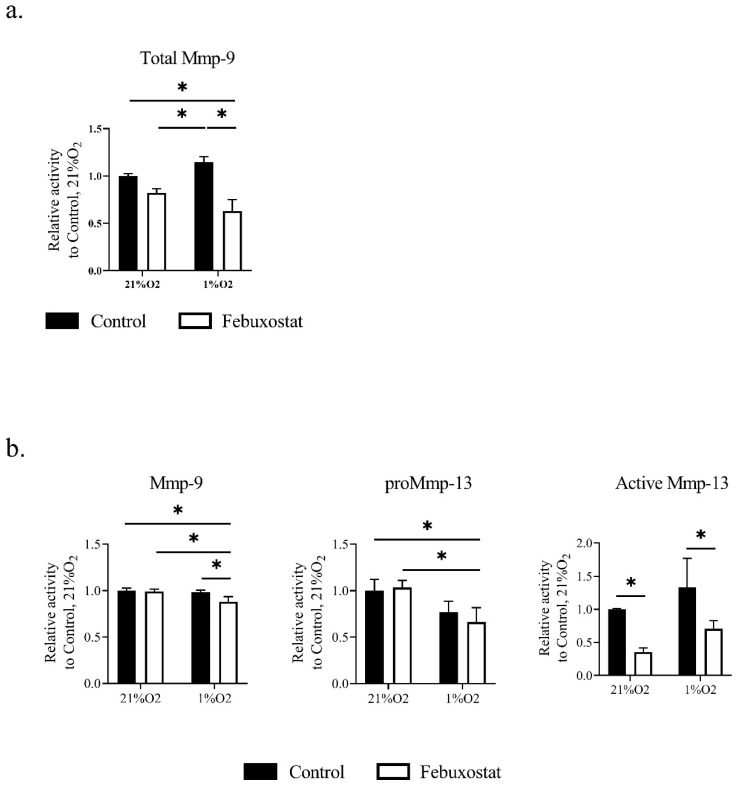
Inhibition of ROS production by XO attenuates gelatinolytic and collagenolytic activity in TEPMs. TEPMs were cultured under normoxic (21% O_2_) and hypoxic (1% O_2_) conditions in the presence of DMSO (control) or 50 μM febuxostat. MMP activity in the supernatants was assayed by in-gel zymography. (**a**) The activities of MMP-9 was assayed by gelatin zymography. (**b**) The activities of MMP-9 and 13 were assayed by collagen zymography. The quantification of MMP activity was performed using a densitometry analytic tool (Supplementary Figure S2). Data are expressed as the means ± SD of three independent experiments. The two-way ANOVA and Tukey’s multiple comparisons test were performed for the statistical analysis. * *p* < 0.05.

It was previously reported that MMP-3 and -10, also known as stromelysin-1 and 2, digest several extracellular matrix proteins but not interstitial collagen. Furthermore, these MMPs participate in proMMP activation for tissue remodeling. Stromelysin-1 (MMP-3) activates gelatinase B (MMP-9) in vascular smooth muscle cells (VSMCs), and contributes to neointima formation [24]. On the other hand, Stromelysin-2 (MMP-10) is expressed in macrophages. *MMP*-10-deficient macrophages reduce collagenolytic activity, particularly MMP-13 [25]. Therefore, to examine the hypothesis that febuxostat attenuates collagenolytic and gelatinolytic activity in macrophages, we measured MMP activity in culture media of macrophages by in-gel zymography [9,26]. The zymographic analysis showed that febuxostat significantly suppressed collagenolytic and gelatinolytic activity in macrophages (Figure 4a,b). These results suggest that inhibition of XO signaling attenuated the activity of collagenase and gelatinase in TEPMs.

### 2.4. XO Inhibitor Suppresses the Rupture of Atherosclerotic Plaques in Mice

**Figure 5:**
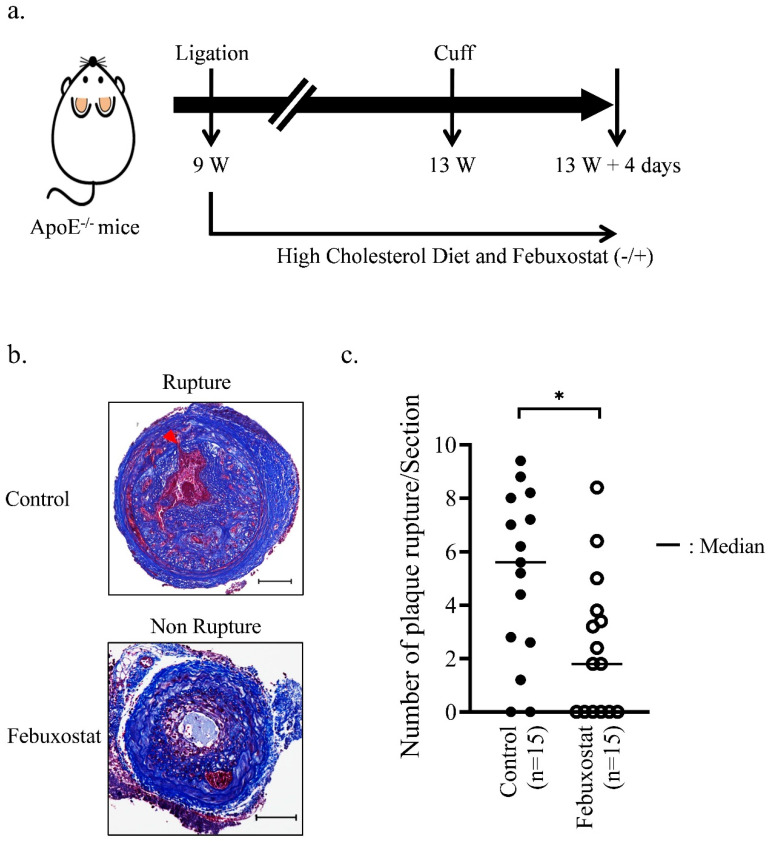
Febuxostat treatment reduces rupture events of the common carotid artery. (**a**) Schematic diagram of the operation for the atherosclerotic plaque rupture model. After the carotid artery ligation, mice were divided into the experimental and the control groups, and then fed a high-cholesterol diet. The experimental group was given drinking water containing 0.015 mg/mL of febuxostat. (**b**) Representative images of pathological alterations in the plaque rupture mouse model. Masson’s trichrome staining were performed using sections of the common carotid artery from *Apoe*−/− male mice fed a high-cholesterol diet for 4 weeks after ligation. Representative photographs show non-ruptured and ruptured vessels. The black bar indicates 100 μm. (**c**) The number of plaque ruptures was quantitatively analyzed by counting the number of internal elastic lamina breaks (arrowhead) in the Fbuxostat group (*n* = 15) and the Control group (*n* = 15). The Mann-Whitney U test was used to compare differences between the control group and Febuxostat group. * *p* < 0.05.

MMP production from macrophages contributes to atherosclerotic plaque instability and rupture [4]. It was examined whether XO signaling contributed to the vulnerability of atherosclerotic plaques using an atherosclerotic plaque rupture mouse model generated by the ligation and cuff placement method (Figure 5a) [27]. Atherosclerotic lesions were formed (Figure 5b) as follows: 15 mg/L febuxostat was continuously administered to these mice via their drinking water for 32 days. The mice were then euthanized to perform histological analyses. As shown in Figure 5c, the average number of plaque rupture lesions was significantly decreased in mice that drank the water containing febuxostat. These findings suggest that XO activity contributed to the instability of atherosclerotic plaques through MMP.

## 3. Discussion

It is well understood that inflammatory macrophages contribute to atherosclerotic plaques’ formation and vulnerability [28]. The microenvironment in atherosclerotic plaques contains various oxidized lipids and cytokines. Macrophages are exposed to these stimuli, resulting in the inflammatory phenotype. These inflammatory macrophages express and secrete pro-inflammatory cytokines and MMPs. Macrophages in atherosclerotic lesions have strong proteolytic activity and cause the vulnerability and rupture of plaques. At the same time, it has also been reported that the macrophage-rich region in human carotid atherosclerotic lesions is a hypoxic environment [20]. Therefore, macrophages within atherosclerotic plaques are exposed to not only inflammatory but also hypoxic environments. However, little is known about whether hypoxic stress on macrophages affects atherosclerotic plaque progression.

The present study reveals that hypoxic stress to macrophages induces the expression of *MMP*-3, -10, and -13 mRNAs, and activates gelatinolytic and collagenolytic activity. It was previously reported that MMP-3, -10, and -13 were involved in the development of atherosclerotic lesions and plaque progression [29,30,31,32]. MMP-3 was one of the first MMPs identified in atherosclerotic plaques, and it is expressed in macrophages, as well as lymphocytes and activated VSMCs [5]. The *MMP*-3 knockout mice showed a reduction in intimal growth caused by the ligation of carotid arteries. MMP-10 expression in the atherosclerotic lesion is mainly localized in macrophage-like cells, and it is necessary for collagenolytic activity. *MMP*-10/*Apoe* double knockout (DKO)mice show decreased atherosclerotic lesion size and plaque calcification. *MMP*-13 mRNA was abundantly expressed in macrophages. *MMP*-13/*Apoe* DKO mice showed the accumulation of collagen in the plaque and resulted in plaque stabilization. These previous reports and the present study suggest that hypoxic signaling in macrophages causes instability and rupture of atherosclerotic plaques by expressing *MMP*-3, -10, and -13.

Intriguingly, the hypoxic induction of *MMP*-3 and -10 mRNAs was dependent on ROS generated by XO, but not HIF-1α, which is known as the central regulator of the hypoxic response. Previous studies have reported that hypoxia activates p38/SAPK2, JNK/SAPK, and Akt/PKB signaling [33,34,35]. In addition, it has been reported that ROS also activates p38, JNK, and Akt kinases [36,37]. Actually, we confirmed that inhibitors of p38 and JNK suppress the hypoxic induction of *MMP*-3, -10, and -13 mRNAs (data not shown). Therefore, we consider that ROS production under the hypoxic condition induces *MMP*-3, -10, and -13 mRNAs via the activation of these kinases.

At the same time, it is known that ROS interacts with the Cys thiol within the catalytic domain of MMPs, which disrupts the interaction with the catalytic Zn^2+^ ion and leads to autocatalytic activation. Previous studies report that ROS activates various proMMPs, including MMP-1, MMP-2, MMP-7, MMP-8, and MMP-9 [38,39,40,41,42,43]. Therefore, there is a possibility that our in-gel zymographic analysis shows the activation of gelatinase and collagenase by not only MMP-3 and -10 but also ROS as the direct effect. Further investigations are needed to elucidate the detailed mechanisms of the hypoxic induction and activation of these *MMP*s.

Moreover, febuxostat significantly inhibited the production of MMP-9 and the activation of MMP-13 even under normoxic conditions. Previous studies reported that ROS activates MMP secretion [44,45]. In general, the mitochondria respiratory chain (MRC) consumes most oxygen in cells, and 2~4% of total oxygen consumption goes toward the generation of ROS instead of ATP synthesis [46]. Therefore, mitochondria constitutively produce ROS. A recent study has reported that inhibition of xanthine oxide reductase (XDH/XO) by febuxostat causes the increment of intracellular ATP by purine salvage pathway and reduces the production of ROS from MRC under normoxic conditions [47]. Thus, febuxostat may affect the production and activation of MMPs by reducing ROS derived from MRC under normoxic conditions. However, future studies are required to uncover the precise mechanism of inhibitory effects of febuxostat on MMP expression without transcriptional regulations.

Using the atherosclerotic plaque rupture mouse model, this study demonstrated that febuxostat administration significantly reduced the number of plaque ruptures. Febuxostat also attenuated collagenolytic (MMP-13) and gelatinolytic activity (MMP-9) in the culture media of macrophages. These results may indicate that the hypoxic environment accelerates atherosclerotic plaque vulnerability and rupture by activating MMPs via XO. Previous clinical trials reported that the administration of allopurinol, a purine inhibitor of XO, was associated with a lower rate of stroke and cardiac events [48,49]. Febuxostat, a nonpurine inhibitor of XO, as well as allopurinol, is used for the management of hyperuricemia in patients with gout [50]. In addition to these beneficial effects, this study suggests that the administration of an XO inhibitor could be useful for suppressing atherosclerotic plaque instability and rupture in patients.

## 4. Materials and Methods

### 4.1. Cell Culture

TEPMs were prepared from 7-week-old C57BL6/J mice by the intraperitoneal injection of 3% thioglycollate media (Fluka, Sigma-Aldrich, St Louis, MO, USA). On day 4, TEPMs were collected by peritoneal lavage with 10 mL of PBS(−) and isolated using the adherent method. TEPMs were seeded onto ϕ6-cm dishes at 4 × 10^6^/dish and grown in RPMI1640 (WAKO, Osaka, Japan) containing 10% fetal bovine serum (Sigma-Aldrich, St Louis, MO, USA) at 37 °C in 5% CO_2_. TEPMs were treated by the following inhibitors at the indicated concentrations in each experiment: *N*-acetyl-l-cysteine (NAC) (10 mM), Mito-TEMPO (50 μM), VAS2870 (2 μM), and febuxostat (50 μM). In all experiments, TEPMs were pretreated with these inhibitors for 1 h before stimulation by hypoxia. NAC, Mito-TEMPO, and VAS2870 were purchased from Sigma-Aldrich (St Louis, MO, USA). Febuxostat was provided by Teijin Pharma Ltd., Tokyo, Japan.

### 4.2. Hypoxia Treatment

Cells were placed in a personal CO_2_ multi-gas incubator (Model APM-30D, ASTEC, Fukuoka, Japan) calibrated to maintain a hypoxic atmosphere (1% O_2_, 5% CO_2_, and 94% N_2_) under a continuous flow of nitrogen. A standard tissue culture incubator was used for a normoxic environment (21% O_2_, 5% CO_2_).

### 4.3. Mouse Models of Atherosclerotic Plaque Rupture

All animal experiments were approved by the Institutional Animal Care Committee of Nagasaki University (approval number: 1701251356). B6.129P2-*Apoe*^tm1Unc^/J (*ApoE*−/−) mice were kindly provided by Dr. J. Aruga (Nagasaki University, Nagasaki, Japan). To generate mouse models of atherosclerotic plaque rupture, 9-week-old male *ApoE*^−/−^ mice were fed a standard diet. The left common carotid artery of these mice was dissected and ligated proximal to the bifurcation under anesthesia. After the ligation, the mice were randomly divided into two groups, including the experimental group (febuxostat) and the normal control group (Control), and fed a high-cholesterol diet (Oriental Yeast, Tokyo, Japan). The experimental group was given drinking water containing 0.015 mg/mL of febuxostat after the ligation. At 4 weeks after the ligation of the left common carotid artery, a polyethylene cuff (length 2 mm) was placed around the left common carotid artery tightly adjacent to the site of ligation. At 4 days after cuff placement, mice were anesthetized and injected intraperitoneally with heparin. Lesions were then obtained after perfusion from the left ventricle using a normal saline solution. The vessels after cuff removal were isolated and fixed in 10% formalin overnight.

### 4.4. Histological and Immunohistochemical Staining

For staining, vessels were fixed in 4% formaldehyde and embedded in paraffin. Specimens were serially sectioned at 5 μm intervals and mounted on three consecutive slides with 60-µm intervals on glass slides. Two slides were individually stained with hematoxylin and eosin (H&E) and Masson’s trichrome stains. Five serial, identically stained slides were selected from the middle part of the cuff placement site of each vessel to count the number of internal elastic lamina breaks. The number of ruptures was taken as the average of 5 sections.

### 4.5. Quantitative Real-Time RT-PCR Analysis

Total RNA was purified from culture cells using the RNeasy Mini Kit (Qiagen, Thermo Fisher Scientific, Inc., Waltham, MA, USA). Complementary DNA (cDNA) was synthesized using the ReverTra Ace^®^ qPCR RT Master Mix (TOYOBO, Osaka, Japan). Quantitative real-time PCR (qPCR) was performed using the THUNDERBIRD^®^ SYBR^®^ qPCR MIX kit (TOYOBO, Osaka, Japan). A melting curve analysis was performed to confirm the detection of a single PCR product. We performed the standard curve method for the quantitative analysis of qPCR. Then, 5, 25, 125, and 625-fold dilutions of a sample were prepared for each gene. The standard curve was drawn from the Ct values of the samples. The expression levels of each sample were calculated using the standard curve. The relative abundance of all genes of interest was normalized to the housekeeping control 18S ribosomal RNA (18S rRNA). The primer sequences of the analyzed genes are shown as follows: *MMP*-3 (NCBI accession number NM_010809.2): forward 5′-TGCAGCTCTACTTTGTTCTTTGA-3′, reverse 5′-AGAGATTTGCGCCAAAAGTG-3′. *MMP-9* (NCBI accession number NM_013599.4): forward 5′-CGACATAGACGGCATCCAG-3′, reverse 5′-CTGTCGGCTGTGGTTCAGT-3′. *MMP*-10 (NCBI accession number NM_019471.3): forward 5′-GAGTCTGGCTCATGCCTACC-3′, reverse 5′-CAGGAATAAGTTGGTCCCTGA-3′. *MMP-12* (NCBI accession number NM_008605.3): forward 5′-TGATGCTGTCACAACAGTGG-3′, reverse 5′-GTAATGTTGGTGGCTGGACTC-3′. *MMP*-13 (NCBI accession number NM_008607.2): forward 5′-TGGACCTTCTGGTCTTCTGG-3′, reverse 5′-ATGGGCAGCAACAATAAACA-3′. *MMP-14* (NCBI accession number NM_008608.4): forward 5′-GAGAACTTCGTGTTGCCTGA-3′, reverse 5′-CTTTGTGGGTGACCCTGACT-3′. *Pgk1* (NCBI accession number NM_008828.3): forward 5′-CTGTGGTACTGAGAGCAGCAAGA-3′, reverse 5′-CAGGACCATTCCAAACAATCTG-3′. *IL6* (NCBI accession number NM_031168.2): forward 5′-GATGGATGCTACCAAACTGGAT-3′, reverse 5′-CCAGGTAGCTATGGTACTCCAGA-3′. *18S rRNA* (NCBI accession number NR_003278.3 forward 5′-GCAATTATTCCCCATGAACG-3′, reverse 5′-GGGACTTAATCAACGCAAGC-3′.

### 4.6. Zymography and Quantification

TEPMs were exposed to hypoxia for 24 h in the presence or absence of febuxostat, and supernatants were collected. Cell supernatants were clarified by brief centrifugation and concentrated using the Amicon Ultra-2, 3-kDa nominal molecular mass cutoff filters (Millipore, Billerica, MA, USA). The protein concentration of supernatants was determined by the BCA Protein Assay kit (Pierce, Thermo Fisher Scientific, Inc.). Equal amounts (5 μg/well) were loaded onto 10% zymogram plus (gelatin) protein gels (Novex; Thermo Fisher Scientific, Inc., Waltham, MA, USA) for the gelatin zymogram or 10% SDS-PAGE containing 0.6 mg/mL rat type I collagen (Gibco, Thermo Fisher Scientific, Inc., Waltham, MA, USA) for the collagen zymogram, and run for 90 min under a constant voltage of 125 V. Gels were incubated in a renaturing buffer (Novex; Thermo Fisher Scientific, Inc., Waltham, MA, USA), followed by incubation at 37 °C for 24 h in developing buffer (Novex; Thermo Fisher Scientific, Inc., Waltham, MA, USA). Gels were stained with the SimplyBlue SafeStain (Invitrogen, Thermo Fisher Scientific, Inc., Waltham, MA, USA). Zymograms were analyzed by densitometry analysis using the ChemiDoc Touch Imaging System (BIO RAD Laboratories Inc., Hercules, CA, USA) (Supplementary Figure S2).

### 4.7. Statistical Analysis

All data are shown as means with SD. Comparisons between two groups were performed using the unpaired *t*-test. *p* values of less than 0.05 were considered significant. Differences among more than two groups were analyzed using one-way or two-way ANOVA followed by Dunn’s multiple comparisons test or Tukey’s multiple comparisons test.

## Supplementary Materials

The following are available online at https://www.mdpi.com/2227-9059/8/11/470/s1.
