# Supplementary material for: Febuxostat, a Xanthine Oxidase Inhibitor, Decreased Macrophage Matrix Metalloproteinase Expression in Hypoxia
Source: Biomedicines. 2020 Nov 3;8(11):470. doi: 10.3390/biomedicines8110470 (PMC7693746; doi:10.3390/biomedicines8110470)
Supplement: Supplementary file 1 [file biomedicines-08-00470-s001.pdf]

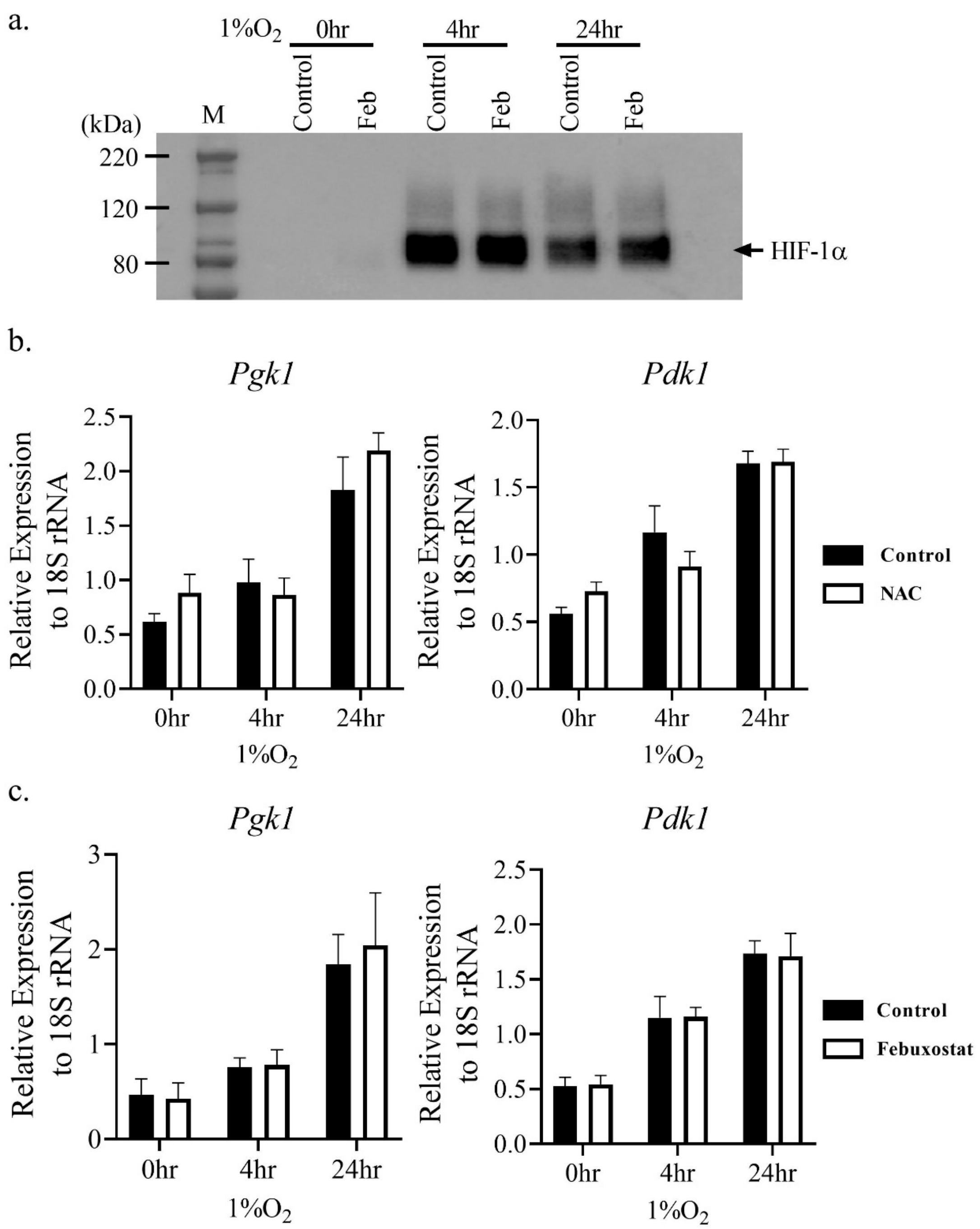

Supplementary Figure 1. Febuxostat, an XO inhibitor, does not affect the HIF-1α signaling pathway. (a) Immunoblot analysis of HIF-1α protein expression in TEPMs treated with febuxostat under hypoxia. Two micrograms of a nuclear extract of TEPMs were loaded onto 10% SDS/PAGE gel. M indicates molecular weight marker. (b) NAC (anti-oxidant) does not inhibit the expression of *Pgk1* and *Pdk1* mRNAs induced by hypoxia. (c) Febuxostat (XO inhibitor) does not inhibit the expression of *Pgk1* and *Pdk1* mRNAs induced by hypoxia. Data are the means and SE of at least three independent experiments.

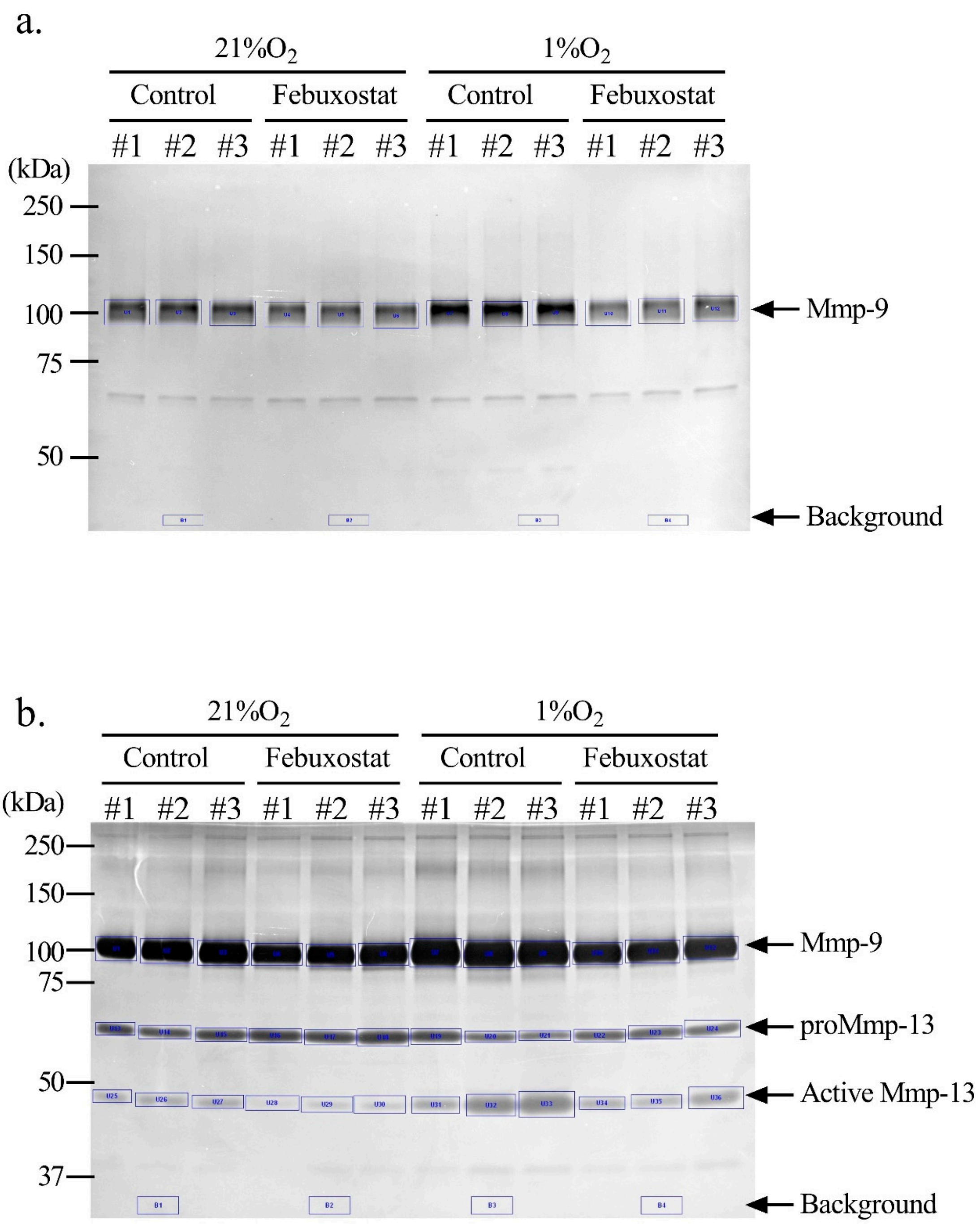

Supplementary Figure 2. Densitometry analysis of gelatin and collagen zymography. Representative images of in-gel zymography are shown. (a) gelatinolytic and (b) collagenolytic activities of the conditioned media were analyzed. Five micrograms of conditioned media were loaded onto a zymogram gel. Squares show how the set of bands from each lane was selected for densitometry.
